# Dietary intake and breast density in high-risk women: a cross-sectional study

**DOI:** 10.1186/bcr1781

**Published:** 2007-10-19

**Authors:** Marilyn Tseng, Celia Byrne, Kathryn A Evers, Mary B Daly

**Affiliations:** 1 Division of Population Science, Fox Chase Cancer Center, 333 Cottman Avenue, Philadelphia, PA 19111, USA; 2 Division of Cancer Genetics and Epidemiology, Lombardi Comprehensive Cancer Center, 3800 Reservoir Road NW, Washington, DC 20007, USA; 3 Department of Diagnostic Radiology, Fox Chase Cancer Center, 333 Cottman Avenue, Philadelphia, PA 19111, USA

## Abstract

**Background:**

Women with a family history of breast cancer may be at higher risk for breast cancer, but few previous studies evaluating diet and breast cancer have focused on such women. The objective of the present study was to determine whether diet, a modifiable risk factor, is related to breast density among women at high genetic risk for breast cancer.

**Methods:**

Women with at least one first-degree or second-degree relative with breast cancer or ovarian cancer participating in the Fox Chase Cancer Center Family Risk Assessment Program completed health history and food frequency questionnaires and received standard screening mammograms. Cranial–caudal mammographic images were classified into the four Breast Imaging Reporting and Data System categories ranging from 'entirely fatty' to 'extremely dense'. Logistic regression analysis using proportional odds models for polychotomous outcomes provided estimates of odds ratios for having a higher category versus a lower category of breast density.

**Results:**

Among 157 high-risk women, breast density was inversely associated with vitamin D intake (odds ratio for third tertile versus first tertile, 0.5; 95% confidence interval, 0.2–1.0). In contrast, intakes above the median level for protein (odds ratio, 3.0; 95% confidence interval, 1.3–6.9) and above the median level for animal protein (odds ratio, 4.3; 95% confidence interval, 1.8–10.3) were associated with higher breast density, but only among women whose family history did not reflect a known familial cancer syndrome or a breast cancer predisposition gene.

**Conclusion:**

For women with a strong family history that was not associated with known cancer syndromes, dietary factors may be associated with breast density, a strong predictor of breast cancer risk. Since women with strong family history are often very motivated to change their lifestyle habits, further studies are needed to confirm whether changes in diet will change the breast density and the subsequent onset of breast cancer in these women.

## Introduction

Women with a family history of breast cancer may be at higher genetic risk for breast cancer because of either recognized deleterious mutations or as yet unidentified low-penetrance alleles. Such women are likely to be especially motivated to modify their lifestyle to reduce their risk for breast cancer. Diet modification is one possible strategy, but few previous studies evaluating diet and breast cancer have focused on women at high genetic risk [1,2].

Breast density, the percentage of total breast area with a mammographically dense appearance, is an informative marker for breast cancer risk because of its strong association with breast cancer [3-5]. Breast density has been shown to predict risk among women with a genetic predisposition for breast cancer [6], including women carrying BRCA1 or BRCA2 mutations [7]. Among the few studies on diet and breast density [8-16], only one specifically included women with a family history of the disease [17]. The objective of the present study was to evaluate associations between dietary intake and breast density among women at potentially high genetic risk for breast cancer. We focused on dietary factors associated with breast density in previous studies conducted among average-risk samples [8-10,12-15,18], as well as on suspected risk factors or protective factors for breast cancer [19]

## Materials and methods

### Study population

The study sample was drawn from 1,313 women enrolled in the Family Risk Assessment Program (FRAP) at Fox Chase Cancer Center in Philadelphia from September 1991 to April 2001. The FRAP was initiated in 1991 to offer education and preventive interventions, and to serve as a research base for studying gene–environment interactions in breast cancer and ovarian cancer. Women are eligible for the FRAP if they have at least one first-degree or second-degree relative with breast cancer or ovarian cancer. Recruitment strategies include referrals from breast cancer patients or ovarian cancer patients at Fox Chase Cancer Center, radio and newspaper advertisements, and physician referrals and self-referrals.

FRAP participants were eligible for the breast density study if they were at least 40 years old. Exclusion criteria included a history of breast augmentation or reduction, a history of prophylactic mastectomy, a history of cancer except nonmelanoma skin cancer, a current or planned pregnancy, current breastfeeding, a weight change of at least 20 lb during the past year, a substantial change in diet over the past year, or no mammogram planned within the study timeframe. Of 510 potentially eligible women invited to participate, 177 women agreed, 153 women declined, 125 women could not be contacted, and 55 women were subsequently found to be ineligible. An additional 13 women were excluded – because their questionnaires were inadequately completed (*n *= 7), because their mammograms could not be obtained (*n *= 5), or because they reported an energy intake >3,500 kcal/day (*n *= 1) – leaving 164 for inclusion in analyses. A comparison of the women included in the analysis with remaining women in the participant pool showed no significant differences in age, ethnicity, level of education, body mass index, dietary intake, or smoking status, but participants were more likely than nonparticipants to have a first-degree relative with breast cancer (74% versus 61%, *P *= 0.008).

### Data collection

Upon enrollment into the FRAP, women completed a health history questionnaire for baseline information on sociodemographic characteristics, family history of cancer, and reproductive factors, including age at menarche. In 1996, detailed questions on pregnancy history were added to the baseline questionnaire. Each year after their baseline, the FRAP participants were also asked to complete a follow-up questionnaire for updated information on pregnancies and new occurrences of cancer in the family.

Pedigrees provided at baseline and updated annually were reviewed by a team of genetic counselors, and were classified as reflecting one of four categories: (1) sporadic, indicated by a single occurrence of cancer diagnosed at any age occurring on one side of the family only; (2) familial, reflecting a pattern of cancers seen in at least one generation but not fitting a known cancer family syndrome; (3) putative hereditary, fitting a hereditary pattern of cancer inheritance or known cancer family syndrome not yet confirmed by genetic testing; or (4) confirmed hereditary, representing inheritance of a cancer predisposition gene confirmed through genetic testing performed on the proband or on a relative. Women in the putative and confirmed hereditary categories (categories (3) and (4)) were grouped together in analyses to reflect the presence of a cancer family syndrome whether based on pedigree analysis or genetic testing. Such pedigrees primarily reflected the presence of a BRCA1 or BRCA2 mutation but also included other cancer family syndromes such as Li–Fraumeni syndrome or Cowden's disease.

All FRAP participants were also asked to complete the Harvard Diet Assessment Form [20] for information on intake frequencies over the previous year of 126 food items and of vitamin and mineral supplement use. The Diet Assessment Form was found to be reasonably valid in a sociodemographically similar sample of women [21].

Participants in the breast density study received a standard screening mammogram at Fox Chase Cancer Center, were measured for weight and height, completed another Diet Assessment Form for their dietary intake for the year previous to the study mammogram, and completed an additional questionnaire for updated information on smoking habits, physical activity, and reproductive factors including pregnancies, breastfeeding, use of hormonal contraceptives or hormone replacement medicine, age at first live birth, and menopausal status. Menopausal status was determined based on whether the participant reported having had a menstrual period within the last year. The study protocol was approved by the Institutional Review Board of the Fox Chase Cancer Center (IRB protocol number 00-803), and all participants gave their written informed consent to participate in the study.

### Breast density assessment

All participants received a standard screening mammogram. For premenopausal women, all efforts were made to schedule mammograms during the follicular phase of each woman's menstrual cycle, when breast tissue is less radiographically dense [22]. Breast density was assessed by the study radiologist (KAE), who was blinded to the identity and other personal characteristics of study subjects, using the Breast Imaging Reporting and Data System: 1 = entirely fatty, 2 = scattered fibroglandular tissue, 3 = heterogeneously dense, and 4 = extremely dense [23].

Results presented below are based on radiological classifications for the cranial–caudal view of a randomly selected side. Consistent with previous work [24], however, left-side and right-side assessments were highly correlated (*r *= 0.97) in the 172 women with mammographic data, and assessments between left and right sides agreed in all but 10 (6%) women. In reproducibility assessments conducted in a randomly selected set of 52 images, we calculated a kappa statistic of 0.97 for the left-side images and 0.89 for the right-side images.

### Data analysis

We used logistic regression analysis for polychotomous outcomes using proportional odds models [25] to estimate the odds ratios. All log odds can be interpreted as the odds for a woman having a higher category versus a lower category for breast density [25]. Dietary factors of interest were calories, total fat and saturated fat, cholesterol, protein, animal protein, carbohydrates, dietary fiber, carotene, folate, calcium, vitamin D, and vitamin E, as well as foods and food groups including meats, fruits and vegetables, tofu, and alcoholic beverages. Nutrient values included both dietary and supplemental sources and were energy-adjusted using the residual method [26]. The odds ratios were estimated for upper tertiles versus lower tertiles of intake; but for infrequently consumed items such as tofu, estimates were for consumption versus nonconsumption. Variables evaluated as potential confounders included age, body mass index, level of education (college graduate or not), age at menarche, number of live births, age at first live birth, ever breastfed, menopausal status, ever use of hormonal contraceptives, ever use of hormone replacement therapy, physical activity (hours per week), smoking status (never, former, current), family history category (sporadic, familial, hereditary), and energy intake. Too few women reported current use of either hormonal contraceptives or hormone replacement therapy to examine their associations with breast density separately from previous use.

Final multivariate models included 157 women with complete covariate data and adjusted for age, body mass index, caloric intake, age at menarche, menopausal status, history of hormone replacement therapy use, and family history category. Results were not materially different when we excluded six women who reported current use of either raloxifene or tamoxifen. Because detailed questions on pregnancy history were not included in the baseline questionnaire until 1996, a large number of women had incomplete data on number of live births and age at first live birth. We therefore ran separate models to examine potential confounding by these factors in the subset of women with complete data for these variables.

The *P *values for a linear trend were obtained for each dietary variable using an ordinal variable representing the scaled median value for each tertile. We examined effect modification by menopausal status using stratified models with each dietary variable dichotomized at the median. The *P *values for interaction were obtained from a model including all women, with a dietary variable x menopausal status interaction term. Because we observed no significant differences in effect estimates by menopausal status, results presented are for premenopausal women and postmenopausal women combined. We used a similar strategy to examine effect modification by family history category, comparing women with a hereditary family history of cancer with women without such a family history. We combined women with sporadic breast cancer family histories and women with familial breast cancer family histories because there were too few women (*n *= 32) with only a family history of sporadic breast cancer. In contrast to women with a hereditary family history of cancer based on pedigrees or genetic testing representing a stronger genetic predisposition to breast cancer, women with sporadic breast cancer family histories and familial breast cancer family histories may represent women with lower-penetrance susceptibility genes that have not yet been identified, but that still places them at higher genetic risk.

## Results

The 157 women in the analysis had a mean ± standard deviation age of 50 ± 8 years; 96% were white, and 73% had at least a college degree. Among the mammographic images included in the analyses, 12.7% (*n *= 20) were classified as entirely fatty, 28.0% (*n *= 44) as having scattered fibroglandular tissue, 34.4% (*n *= 54) as heterogeneously dense, and 24.8% (*n *= 39) as extremely dense. Having denser breasts was associated with younger age, lower body mass index, older age at menarche, smaller number of live births, older age at first live birth, and a history of having used hormone replacement therapy (Table 1). Menopausal status was inversely associated with breast density, but not significantly after adjustment for age. Level of education, current smoking, and a hereditary family history of breast cancer showed nonsignificant positive associations with breast density. We observed no associations with breast density for breastfeeding, history of oral contraceptive use, or physical activity.

**Table 1:** Characteristics of women by breast density category, and odds ratios for association with denser breast category (*n *= 157)

Characteristic	Entirely fatty (*n *= 20)	Heterogeneously dense (*n *= 44)	Scattered fibroglandular tissue (*n *= 54)	Extremely dense (*n *= 39)	Age-adjusted odds ratio (95% confidence interval)^a^
Age (years)	54.1 ± 1.0	53.9 ± 0.9	48.7 ± 0.6	47.4 ± 0.7	0.4 (0.3–0.6)^b^
College graduate (%)^c^	55	72	72	84	1.5 (0.8–3.0)
Body mass index (kg/m^2^)	33.3 ± 7.1	28.3 ± 6.1	24.8 ± 4.4	23.8 ± 3.0	0.8 (0.8–0.9)
Age at menarche (years)	12.4 ± 1.3	12.3 ± 1.3	12.5 ± 1.4	13.1 ± 1.8	1.3 (1.1–1.6)
Number of live births^c^	3.1 ± 1.4	2.5 ± 1.6	2.6 ± 0.9	1.9 ± 1.0	0.8 (0.6–1.0)
Age at first birth (years)^c^	23.7 ± 5.9	25.6 ± 4.1	27.4 ± 5.8	29.5 ± 5.1	1.1 (1.0–1.2)
Ever breastfed^c^	30	72	63	75	1.0 (0.4–2.7)
Postmenopausal	65	66	33	28	0.5 (0.3–1.2)
Ever used hormonal contraceptives	45	41	63	37	0.8 (0.5–1.4)
Ever used hormone replacement medicine^d^	85	45	56	36	0.4 (0.2–1.0)
Weekly physical activity (hours)	2.9 ± 2.9	3.6 ± 3.0	3.5 ± 2.9	2.9 ± 2.5	1.0 (0.8–1.1)
Smoking status					
∘ Never smoked	50	49	57	61	1.0
∘ Former smoker	45	49	39	26	0.6 (0.3–1.1)
∘ Current smoker	5	2	4	13	2.0 (0.5–7.6)
Family history of breast cancer					
∘ Negative or sporadic	15	30	22	10	1.0
∘ Familial	35	45	33	33	1.3 (0.6–2.9)
∘ Hereditary	50	25	44	56	1.8 (0.8–4.0)

Characteristics data presented as the mean ± standard deviation or as the percentage. ^a^Odds ratios adjusted for age except for the age variable. ^b^Odds ratio for 10-year age increment. ^c ^Because of missing responses, analyses included only 155 women for education level, 125 women for number of live births, 120 parous women for age at first live birth, and 74 parous women for breastfeeding. ^d^Analysis conducted among 70 postmenopausal women.

Breast density was significantly inversely associated with intake of vitamin D (Table 2); women in the highest tertile of vitamin D intake were one-half as likely to have higher breast density as women in the lowest tertile (odds ratio, 0.5; 95% confidence interval, 0.2–1.0). We also observed a suggestive but nonsignificant inverse association for folate (odds ratio, 0.5 for highest tertile versus lowest tertile; 95% confidence interval, 0.2–1.1; trend *P *= 0.09). Breast density was not associated with any other nutrients of interest (Table 2) or with any foods of interest (meats, fruits and vegetables, tofu, alcoholic beverages – results not shown) among all participants combined.

**Table 2:** Adjusted odds ratios^a ^and 95% confidence intervals by tertile of nutrient intake (*n *= 157)

Nutrient	Tertile 1	Tertile 2	Tertile 3	Trend *P *value^b^
Energy (kcal)				
∘ Median per day	1,357	1,807	2,444	
∘ Odds ratio (95% confidence interval)	1.0	1.6 (0.7–3.3)	0.9 (0.4–2.0)	0.79
Total fat (g)				
∘ Median per day	55.5	60.8	73.1	
∘ Odds ratio (95% confidence interval)	1.0	1.9 (0.9–4.2)	1.5 (0.7–3.1)	0.33
Saturated fat (g)				
∘ Median per day	18.7	22.4	27.2	
∘ Odds ratio (95% confidence interval)	1.0	0.9 (0.4–1.9)	0.9 (0.4–1.9)	0.70
Cholesterol (mg)				
∘ Median per day	200	221	305	
∘ Odds ratio (95% confidence interval)	1.0	1.6 (0.7–3.6)	1.2 (0.5–2.7)	0.72
Protein (g)				
∘ Median per day	76.0	76.0	93.4	
∘ Odds ratio (95% confidence interval)	1.0	1.0 (0.5–2.2)	0.9 (0.4–2.0)	0.87
Animal protein (g)				
∘ Median per day	49.0	51.0	68.4	
∘ Odds ratio (95% confidence interval)	1.0	1.0 (0.5–2.3)	1.1 (0.5–2.3)	0.87
Carbohydrates (g)				
∘ Median per day	174.0	228.5	304.6	
∘ Odds ratio (95% confidence interval)	1.0	0.7 (0.3–1.5)	0.9 (0.4–1.9)	0.79
Fiber (mg)				
∘ Median per day	12.4	18.5	27.2	
∘ Odds ratio (95% confidence interval)	1.0	0.5 (0.2–1.1)	0.7 (0.3–1.5)	0.57
Carotene (IU)				
∘ Median per day	4,969	8,621	17,714	
∘ Odds ratio (95% confidence interval)	1.0	0.5 (0.3–1.2)	0.7 (0.3–1.5)	0.51
Folate (mcg)				
∘ Median per day	344	650	943	
∘ Odds ratio (95% confidence interval)	1.0	0.7 (0.3–1.5)	0.5 (0.2–1.1)	0.09
Calcium (mg)				
∘ Median per day	766	1,271	1,871	
∘ Odds ratio (95% confidence interval)	1.0	1.4 (0.6–3.0)	1.0 (0.5–2.1)	0.83
Vitamin D (IU)				
∘ Median per day	164	463	737	
∘ Odds ratio (95% confidence interval)	1.0	0.7 (0.3–1.5)	0.5 (0.2–1.0)	0.05
Vitamin E (mg)				
∘ Median per day	6.8	40.9	436.4	
∘ Odds ratio (95% confidence interval)	1.0	0.4 (0.2–1.0)	0.6 (0.3–1.2)	0.37

^a^Models adjusted for age, body mass index, caloric intake, age at menarche, menopausal status, hormone therapy use, and family history category. ^b^*P *value for trend obtained by including in the model a variable representing the median value for each tertile.

In stratified analyses, we found evidence for effect modification by family history category (Table 3). Whereas protein intake and animal protein intake were not significantly associated with breast density among women with a hereditary pattern of family history of breast cancer, these nutrients were positively associated with breast density among women with sporadic or other familial histories. The odds ratios for women with intake above the median were 3.0 (95% confidence interval, 1.3–6.9) and 4.3 (95% confidence interval, 1.8–10.3) for protein and animal protein, respectively. These results were not meaningfully different when we controlled for number of live births and age at first live birth in the 120 women with complete data (results not shown).

**Table 3:** Adjusted odds ratios^a ^and 95% confidence intervals for dichotomized dietary intake^b ^by family history category

	Sporadic or familial family history (*n *= 90)		Hereditary family history (*n *= 67)	
	
	Median (g)	Odds ratio (95% confidence interval)	Median (g)	Odds ratio (95% confidence interval)
Protein				
∘ Low	76.2	1.0	77.6	1.0
∘ High	93.5	3.0 (1.3–6.9)	91.0	0.4 (0.1–1.4)
∘ *P *value for interaction^c^	<0.001			
Animal protein				
∘ Low	51.6	1.0	49.0	1.0
∘ High	66.4	4.3 (1.8–10.3)	69.6	0.5 (0.1–1.5)
∘ *P *value for interaction	<0.001			

^a^Models adjusted for age, body mass index, caloric intake, age at menarche, menopausal status, hormone therapy use, and vitamin D intake. ^b^Dichotomized at median value. ^c^*P *value for interaction was obtained by including in the model of a dichotomized dietary variable x family history category interaction term.

## Discussion

In this broad exploration of dietary factors, we found that only vitamin D intake was inversely associated with breast density in a sample of women enrolled in a high-risk program for breast cancer. Protein and animal protein were positively associated with breast density, but only among women not classified as having the hereditary familial cancer patterns.

Our results are consistent with recent work showing an inverse association between vitamin D and breast density [14,18], as well as studies suggesting a protective effect of vitamin D against breast cancer [27]. A previous study observed an inverse association of vitamin D with breast density only among premenopausal women [18], and primarily among premenopausal women with higher levels of insulin-like growth factor 1 or of insulin-like growth factor binding protein 3 [28]. In the present study, we saw no difference in effect by menopausal status. Vitamin D may lower breast density through the antiproliferative and proapoptic effects of its biologically active form, 1,25-dihydroxyvitamin D, or through modulation of the immune system [27,29]. Notably, most tissues and cells, including those in the breast, have vitamin D receptors and are also able to metabolize circulating 25-hydroxyvitamin D to 1,25-dihydroxyvitamin D, allowing for local, cellular effects in the breast tissue [29].

Whether the inverse association we observed is truly attributable to a protective effect of dietary vitamin D, however, remains unclear. We did not assess sunlight exposure, another important determinant of vitamin D status, nor did we have serum samples available to assess the relationship of breast density either with 25-hydroxyvitamin D as an indicator of vitamin D status or with 1,25-dihydroxyvitamin D. Of two previous studies that examined 25-hydroxyvitamin D in relation to breast density, one study saw a protective effect [30] while the other study did not [30]. In additional analyses we explored whether breast density might be more strongly associated with major dietary sources of vitamin D, such as dairy or fish, but we observed no clear associations (results not shown).

Our results are also consistent with previous studies that found positive associations of breast density with protein or meat intake [11,12,31]. Animal protein intake may increase breast density by increasing circulating levels of insulin-like growth factor-1 [32,33], which has been linked to higher breast density [34] and higher breast cancer risk [35-37] among premenopausal women. In the present study, the associations for protein and animal protein were limited to women without a hereditary family history of breast cancer. Classification of a hereditary family history of breast cancer in our sample primarily represented the presence of a BRCA1 mutation or a BRCA2 mutation. While this effect modification may be a chance finding, it may also suggest that, in such women, the same genetic factors that predispose to higher breast cancer risk increase the risk for dense breasts as well [38], and that genetic effects can overwhelm the effects of reduced dietary protein. For example, if BRCA1 exerts downstream effects on transcription, expression, or circulating levels of members of the insulin-like growth factor system as suggested by recent work [39-41], then breast tissue may be denser among BRCA1 mutation carriers [38] regardless of their level of protein intake.

Other dietary factors may affect breast density by affecting estrogen levels or by directly affecting breast tissue morphology [8,10,14] – but only limited, indirect evidence exists to support these mechanisms, and previous study findings with respect to other dietary factors have been inconsistent. In cross-sectional analyses, breast density has been both positively associated [8,9,11,31] and inversely associated [13,17] with total and saturated fat intake. Findings for soy and isoflavones have been similarly mixed [13,15,16,42,43]. Among other nutrients, higher breast density has been related to alcohol intake [17,44,45] and inversely related to carotenoid, fiber, and calcium [8,9,14,18,45,46].

Previous studies have observed a protective effect of folate against breast cancer [47], possibly because of its role in DNA methylation, synthesis, and repair [48], although breast density studies have not supported this [17,45]. In our sample we observed only a suggestive, nonsignificant inverse association. A protective effect of folate may be more pronounced among women with high alcohol intake [47], and alcohol intake in our sample was generally low (median 1.1 g/day alcohol). While too few women reported even moderate alcohol intake to examine folate in this group separately, when we excluded nine women with alcohol intake >15 g/day the inverse association for folate was slightly attenuated (trend *P *= 0.27).

Difficulties in assessing breast density may contribute to the inconsistent results of studies on diet and breast density. Most studies, including the current analysis, defined breast density in categories of percentage density. While the method is more subjective than a computer-assisted method [49], the Breast Imaging Reporting and Data System categories are nevertheless a strong predictor of breast cancer risk [50]. Some workers have suggested that absolute size of the area of density is the more relevant measure [51], but only four previous studies presented results for dense area size [11,15,16,42] – three of which focused specifically on soy or isoflavones [15,16,42].

The relevant time period of exposure is also unclear. Women randomized to a low-fat, high-carbohydrate diet for 2 years as part of the Canadian Diet and Breast Cancer Prevention Study Group experienced a greater decrease in area of breast density relative to a nonintervention group [10,11], suggesting that recent, relatively short-term dietary exposures can affect breast density and possibly breast cancer risk, at least in premenopausal women or perimenopausal women. In contrast, a 2-year soy intervention had no effect on breast density change over time, although soy consumption in early life and adulthood did appear to influence density change [16].

A limitation of the present study is its relatively small sample size, which limited our ability to detect significant associations and effect modification by menopausal status. Our sample is unique, however, in that all women had a family history of breast cancer. Moreover, over 40% of them had a family history indicating the presence of a high-penetrance cancer predisposition gene, or had been tested to confirm it.

Examining only the recent diet may additionally have limited our ability to evaluate the role of dietary intake in relation to breast density. Findings were not different when we used the average value of dietary intake reported at enrollment into the FRAP and that reported for the current breast density study, with a median time interval between the two administrations of 4.5 years, but no other dietary information was available to assess effects of longer-term dietary intake on breast density. Data were incomplete for the number of live births and for age at first live birth; but despite their significant associations with breast density, these two variables were not important confounders among the 120 women not missing this information.

Our findings may be biased if a woman's knowledge of her breast density, or some characteristic related to her breast density, influenced her reporting of her dietary intake, or changed her intake so that her current report was not reflective of her long-term diet. For example, women with higher breast density may have been more likely to perceive themselves at higher risk for breast cancer and to modify their diets accordingly. We saw no differences, however, in either breast density or dietary intake by perception of risk for breast cancer reported at baseline (results not shown).

Because of the large number of dietary factors explored in this analysis, our findings should be interpreted with caution, warrant further investigation into the etiologic mechanism and warrant confirmation in larger hypothesis-based and pathway-based studies.

Finally, our findings may be limited in their ability to be generalized to other populations. Participants were drawn from participants in a high-risk program, and, even among these women, those with a lesser family history of breast cancer were underrepresented in our sample. Nevertheless, the availability of detailed pedigrees is a significant strength of the study as it allowed us to classify women on the basis of their family history of breast cancer in more detail than has been conducted previously, and hence to distinguish women at the highest genetic risk for breast cancer. Our study offers suggestive findings that should be confirmed in a larger sample representing a range of genetic and familial risk for breast cancer, in order to clarify the extent to which diet differences might be related to breast density, and whether effects differ by genetic susceptibility. Future studies might extend these findings to see whether changes in dietary intake would be associated with a change in breast density and a subsequent change in breast cancer risk.

## Conclusion

In summary, in the present broad exploration of dietary factors in women enrolled in a high-risk program for breast cancer, we found that only vitamin D intake was inversely associated with breast density. Protein and animal protein intake were positively associated with breast density, but only among women without a hereditary family history of breast cancer. Our latter, tentative finding suggests that some dietary modification strategies may be more effective in reducing breast density and hence breast cancer risk in women with a family history of breast cancer suggestive of lower-penetrance susceptibility genes, but this requires confirmation in a larger sample representing a range of genetic risks for breast cancer.

## Abbreviations

FRAP = Family Risk Assessment Program.

## Competing interests

The authors declare that they have no competing interests.

## Authors' contributions

MT initiated the research project, conducted the statistical analyses, and drafted the manuscript. CB provided input on subsequent revisions of the manuscript. KAE conducted the qualitative breast density assessments. MBD is the Principal Investigator for the FRAP, contributed to the design of this study, and provided input on the manuscript. All authors read and approved the final manuscript.
